# The W100 pocket on HIV-1 gp120 penetrated by b12 is not a target for other CD4bs monoclonal antibodies

**DOI:** 10.1186/1742-4690-9-9

**Published:** 2012-01-27

**Authors:** Maria J Dueñas-Decamp, Olivia J O'Connell, Davide Corti, Susan Zolla-Pazner, Paul R Clapham

**Affiliations:** 1 Center for AIDS Research, Program in Molecular Medicine, University of Massachusetts Medical School, Worcester, Massachusetts, MA 01605; 2 Humabs Biomed SA, Bellinzona, CH-6500, Switzerland; 3 Department of Pathology, New York University Langone School of Medicine, New York, NY 10016

**Keywords:** HIV, envelope, gp120, CD4 binding site, neutralization

## Abstract

**Background:**

The conserved CD4 binding site (CD4bs) on HIV-1 gp120 is a major target for vaccines. It is a priority to determine sites and structures within the CD4bs that are important for inclusion in vaccines. We studied a gp120 pocket penetrated by W100 of the potent CD4bs monoclonal antibody (mab), b12. We compared HIV-1 envelopes and corresponding mutants that carried blocked W100 pockets to evaluate whether other CD4bs mabs target this site.

**Findings:**

All CD4bs mabs tested blocked soluble CD4 binding to gp120 consistent with their designation as CD4bs directed antibodies. All CD4bs mabs tested neutralized pseudovirions carrying NL4.3 wild type (wt) envelope. However, only b12 failed to neutralize pseudoviruses carrying mutant envelopes with a blocked W100 pocket. In addition, for CD4bs mabs that neutralized pseudovirions carrying primary envelopes, mutation of the W100 pocket had little or no effect on neutralization sensitivity.

**Conclusions:**

Our data indicate that the b12 W100 pocket on gp120 is infrequently targeted by CD4bs mabs. This site is therefore not a priority for preservation in vaccines aiming to elicit antibodies targeting the CD4bs.

## Findings

**Figure 1:**
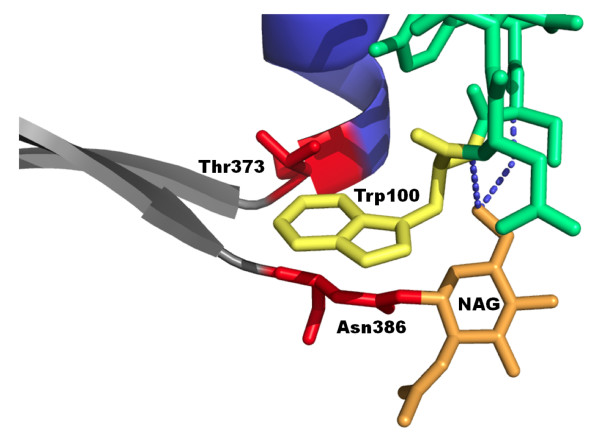
**Proximal gp120 residues T373 and N386 (red) surround the pocket penetrated by the organic rings of b12's W100 (yellow)**. The longer side chain of R373 in combination with the glycan (orange) at N386 may block the pocket and prevent b12 (green) binding.

The conserved CD4 binding site (CD4bs) on HIV-1 gp120 is a major target for the development of vaccines that aim to elicit neutralizing antibodies effective against diverse HIV-1 strains. It is, therefore, important to define sites and structures within the CD4bs that will need to be preserved in vaccines for the induction of neutralizing antibodies. The CD4 binding site (CD4bs) monoclonal antibody (mab), b12, targets a pocket on HIV-1 gp120 as part of its binding site. Thus, the organic rings of b12 W100 penetrate the pocket located immediately downstream from the CD4 binding loop (Figure 1). We showed previously that the presence of a combination of an arginine at residue 373 and a glycan at N386 appears to block the pocket and confer robust resistance to b12 for all five primary HIV-1 envelopes tested [1]. Even the highly sensitive envelope of the T-cell line adapted NL4.3 strain became resistant when carrying the R373/N386 glycan combination. Single substitutions at 373 or that abrogate the glycan at N386 also affect sensitivity to b12 neutralization (our unpublished data and refs [2,3]). However, these changes (in the absence of the R373/N386 glycan combination) are frequently modest and envelope dependent. Here, we have investigated whether the combination of an arginine at residue 373 and a glycan at N386 (which confers resistance to b12) affects the sensitivity of neutralization by other CD4bs mabs.

**Figure 2:**
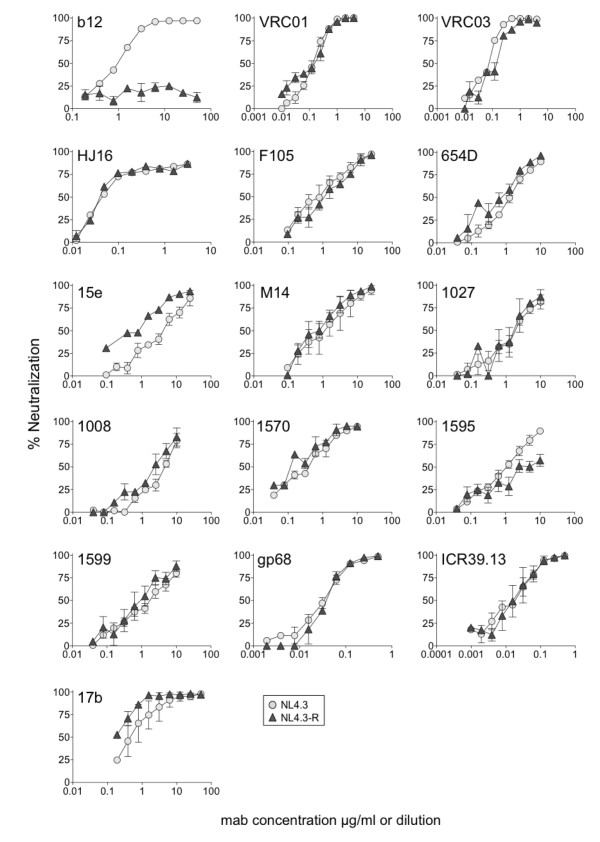
**Neutralization of pseudovirions carrying either NL4.3wt or NL4.3 T373R envelopes**. NL4.3 T373R carries a blocked W100 pocket. Env+ pseudovirions were treated with serial dilutions of each mab and residual infectivity evaluated on HeLa TZM-bl cells using luminescence readouts [8]. Mab dilutions are recorded as μg/ml except for gp68 and ICR39.13, which are hybridoma supernatant dilutions. Each data point shown was obtained from averaging readings obtained from two independent neutralization tests each with duplicate points.

We investigated 15 mabs that block sCD4 binding to gp120 including the potent neutralizing human mabs, b12 [4], HJ16 [5], VRC01 [6,7] and VRC03 [7] (Table 1). Mabs were selected for testing based on two criteria. First, we included mabs previously defined as targeting the CD4bs by their capacity to block gp120: CD4 binding or by crystallization as a complex with gp120. Second, we used CD4bs mabs that were available in sufficient quantities for the neutralization assays described. These included mabs from the NIH AIDS Reagent Program, the UK Centre for AIDS Reagents, the Vaccine Research Center, NIH and from other sources (Table 1). We first confirmed that each of the mabs under investigation blocked sCD4 binding to recombinant gp120 in ELISA assays (Additional File 1: Figure 1). We next tested the capacity of each mab to neutralize NL4.3 wt and NL4.3 T373R (which combines R373 with the glycan already present at N386). NL4.3 is ideal for investigating whether mutation of the W100 pocket affects neutralization since it is highly sensitive to b12 and to each of the CD4bs mabs investigated here. Neutralization assays were done using pseudovirions carrying envelopes from NL4.3wt and NL4.3 T373R (NL4.3-R). HeLa TZM-bl cells were used as targets, and residual infectivity was assessed by measuring luciferase activity [8]. We found that neutralization of NL4.3 by each of the mabs was unaffected or only weakly affected by the R373/N386 glycan combination (Figure 2). Briefly, NL4.3-R appeared marginally more sensitive to mab 15e, yet modestly more resistant to 1595. In addition, the T373R/N386 glycan combination conferred increased sensitivity to the CD4i mab 17b, perhaps indicating a modest shift in envelope conformation towards the CD4-bound form [9].

**Table 1:** CD4 binding site monoclonal antibodies investigated

Mab	Species	Epitope	Source	**Ref**.
b12	human	CD4bs	Polymun Sci. GmbH	[4]
VRC01			NIH VRC^1^.	[6]
VRC03			NIH VRC.	
HJ16			Humabs Inc.	[5]
F105			NIH AIDS Rea. Pr.^2^	[19]
654-D			NIH AIDS Rea. Pr.	[20]
15e			UK/EU CFAR^3^	[21]
M14			UK/EU CFAR	[22]
Gp68			UK/EU CFAR	[23]
1027			Dr Zolla-Pazner	
1008			Dr Zolla-Pazner	
1570			Dr Zolla-Pazner	[24]
1595			Dr Zolla-Pazner	[24]
1599			Dr Zolla-Pazner	[24]

ICR39.13	rat		UK/EU CFAR	[25]

17b	human	CD4i	NIH AIDS Rea. Pr.	[9]

1. NIH Vaccine Research Center.

2. NIH AIDS Reagent Program.

3. Programme EVA Centre for AIDS Reagents, UK.

**Figure 3:**
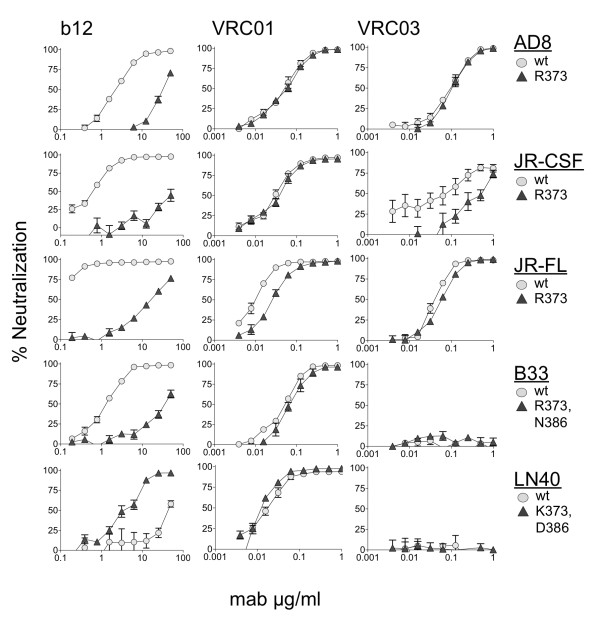
**Neutralization of pseudovirions carrying primary HIV-1 envelopes with and without mutated W100 pockets**. For AD8, JR-CSF and JR-FL, a single substitution introduced R373 to combine with the N386 glycan already present in these envelopes to form the R373/N386 glycan combination to block the W100 pocket. The wild type LN40 envelope already carries the R373/N386 glycan combination and was mutated to change both these residues to K373/D386. The reciprocal substitutions in B33 converted K373/D386 to R373/N386. All envelopes carrying a putative closed W100 pocket were substantially more resistant to mab b12, but remained sensitive to VRC01 and VRC03. Some modest shifts in sensitivity were observed and are discussed in the main text. Each data point shown was obtained from averaging readings obtained from two independent neutralization tests each with duplicate points.

The NL4.3 envelope is derived from a T-cell line adapted HIV-1 and may not accurately represent the structures of primary envelopes *in vivo*. We, therefore, evaluated whether mutation of the W100 pocket affected the sensitivity of primary envelopes. For this experiment, we used AD8 [10], JR-CSF, and JRFL [11] envelopes that were derived from limited culture primary isolates as well as LN40 and B33 that were amplified directly from lymph node and brain tissue of an AIDS patient, respectively [12]. We tested the sensitivity of envelopes with and without a mutated W100 pocket to neutralization by the potent and broad CD4bs mabs, VRC01 and VRC03 and compared with sensitivity to b12 (Figure 3). For AD8, JR-CSF and JR-FL, the mutated envelopes carried a single substitution to R373 to make the R373/N386 glycan combination. For B33, a double substitution introduced R373/N386; while for LN40 (which naturally carries R373/N386), we substituted K373/D386 to reflect the sequence of the B33 envelope. For all five of these primary envelopes, the presence of R373/N386 conferred a dramatic reduction in sensitivity to b12 as expected (Figure 3, left panels). In contrast, for both VRC01 and VRC03, a mutated W100 pocket failed to confer more than minor shifts in neutralization sensitivity. The mutated JR-FL envelope conferred a small shift to increased resistance to VRC01, while JR-CSF became modestly more resistant to VRC03. In contrast, B33 and LN40 wt and mutant envelopes were resistant to VRC03. Together, our data show that the combination of R373 and the N386 glycan does not confer more than modest effects on neutralization by 14 CD4bs mabs contrasting with robust resistance consistently conferred for b12.

Recently, further CD4bs mabs, e.g. 3BNC60 [13] and VRC-PG04 [14] have been reported [13-15]. Like VRC01, VRC03, and HJ16 (studied here), they were derived from so called 'elite neutralizers', HIV-1+ subjects who carried potent neutralizing antbodies active against diverse viral strains. These new mabs are highly potent and have binding specificities that focus on similar gp120 residues to that of VRC01 [6], which (as confirmed here) does not penetrate the W100 pocket on gp120. The crystal structure of mab 3BNC60 verifies a structure where predicted gp120 contact residues are conserved with those of VRC01 [13], while a structure of mab VRC-PG04 complexed with a gp120 core shows the same for that mab and confirms no interaction with the W100 pocket on gp120. Diskin *et al. *also reported that NIH45-46 conferred even more potent neutralization than VRC01. However, the increased potency of this mab was due to increased contact with the gp120 inner domain and bridging sheet determinants without targeting the W100 pocket [16].

In summary, we investigated whether the W100 pocket on gp120 (a critical target for mab b12) is required for HIV-1 neutralization by other CD4bs mabs. We found that neutralization of pseudovirions carrying primary or T-cell line adapted HIV-1 envelopes was either unaffected or only marginally shifted by the presence of a mutated W100 pocket. These observations contrasted with neutralization by b12, which was severely curtailed by the presence of a mutated W100 pocket for all six HIV-1 envelopes tested. Our data indicate that the W100 pocket on gp120 is not a frequent target among CD4bs mabs and is not required for potent neutralization of diverse primary strains of HIV-1 via the CD4bs.

## Availability of supporting data

The data sets supporting the results of this article are available in the Dryad repository http://dx.doi.org/10.5061/dryad.66h5g23t.

## List of abbreviations

None.

## Competing interests

The authors declare that they have no competing interests.

## Authors' contributions

MJD-D helped conceive the study, carried out the neutralization assays and helped write the manuscript. OJO'C carried out the ELISAs and helped interpret their results. DC provided mab, HJ16, and critical discussion of the data. SZ-P provided several mabs and critical discussion of the data and manuscript. PRC conceived the study and wrote the manuscript. All authors read and approved the final manuscript

## Supplementary Material

Additional file 1 **Figure **1. Inhibition of CD4 binding to gp120. ELISAs were carried out to confirm that CD4bs mabs blocked sCD4 binding to gp120. For human mabs (except for gp68), serial (0-8 μg/ml) antibody dilutions were added to saturating immobilized amounts of IIIB gp120 (Immunodiagnostics Inc.) captured with a sheep anti-gp120 C-terminal peptide (Alto Bio Reagents Inc.). Appropriate sCD4 dilutions were then added and bound sCD4 detected using mouse mab, OKT4, followed by an anti-mouse IgG-HRP conjugate (Thermo Scientific Inc.). Mabs gp68 and ICR39.13 were in the form of hybridoma culture supernatant and were added at 1:2 to 1:16 dilutions. ICR39.13 is a rat antibody and was evaluated by competition with PRO542 (CD4-IgG) [17], followed by and ant-human IgG-HRP conjugate. All mabs designated as binding the CD4bs blocked sCD4 binding to recombinant gp120. Most blocked sCD4 highly efficiently, although neither VRC03 nor F105 was particularly effective. Since the structures of VRC03 and F105 bound to gp120 irrevocably prove their specificity for the CD4bs [14,18], the reasons for weaker sCD4 inhibition are unclear. We also tested the CD4i mab, 17b, as a control. As expected, this mab failed to block sCD4 binding to gp120. Click here for file
