# Supplementary figures and images for: The W100 pocket on HIV-1 gp120 penetrated by b12 is not a target for other CD4bs monoclonal antibodies
Source: Retrovirology. 2012 Jan 27;9:9. doi: 10.1186/1742-4690-9-9 (PMC3292835; doi:10.1186/1742-4690-9-9)

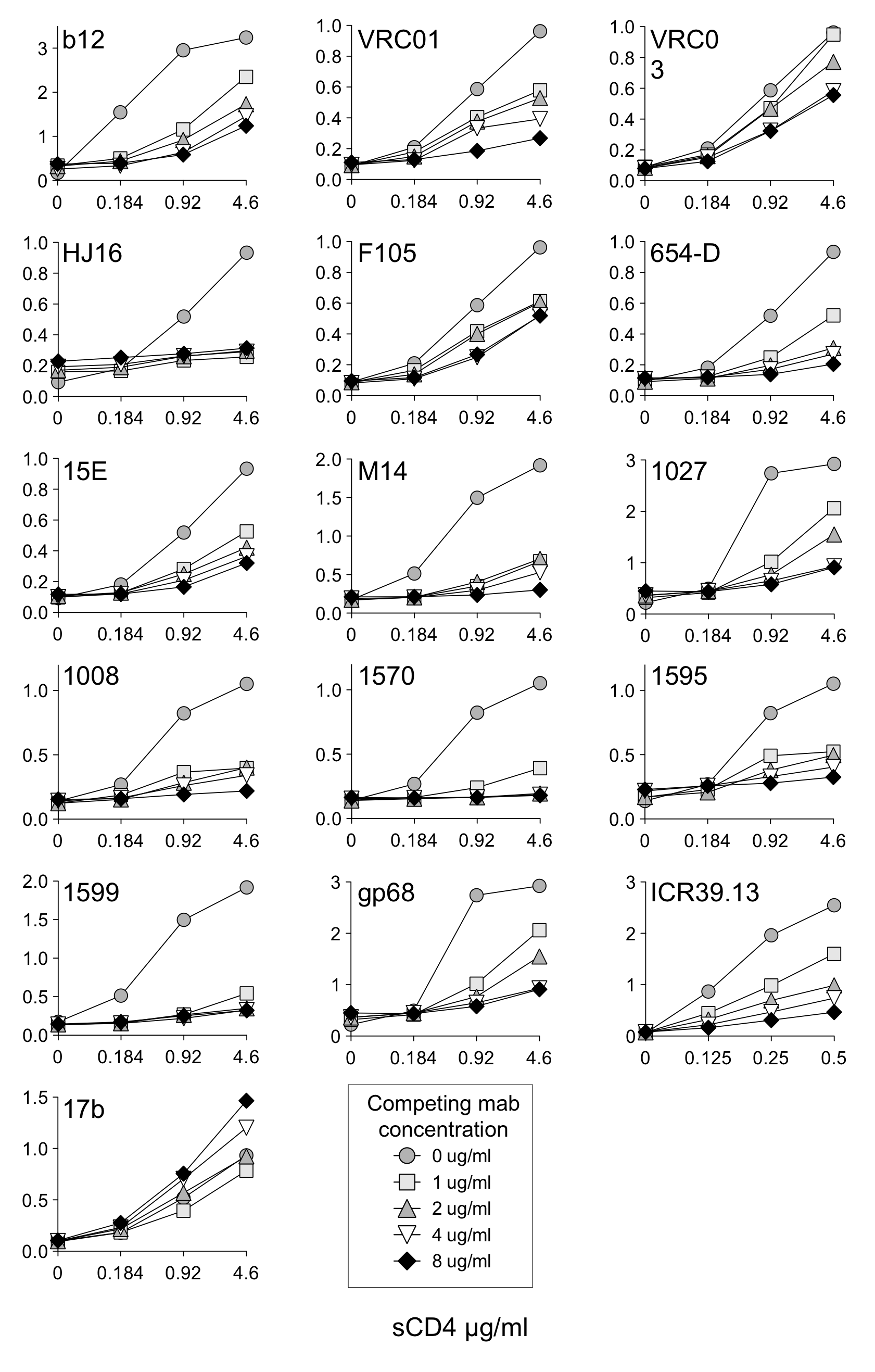

Supplement: Additional file 1 — Figure 1. Inhibition of CD4 binding to gp120. ELISAs were carried out to confirm that CD4bs mabs blocked sCD4 binding to gp120. For human mabs (except for gp68), serial (0-8 μg/ml) antibody dilutions were added to saturating immobilized amounts of IIIB gp120 (Immunodiagnostics Inc.) captured with a sheep anti-gp120 C-terminal peptide (Alto Bio Reagents Inc.). Appropriate sCD4 dilutions were then added and bound sCD4 detected using mouse mab, OKT4, followed by an anti-mouse IgG-HRP conjugate (Thermo Scientific Inc.). Mabs gp68 and ICR39.13 were in the form of hybridoma culture supernatant and were added at 1:2 to 1:16 dilutions. ICR39.13 is a rat antibody and was evaluated by competition with PRO542 (CD4-IgG) [17], followed by and ant-human IgG-HRP conjugate. All mabs designated as binding the CD4bs blocked sCD4 binding to recombinant gp120. Most blocked sCD4 highly efficiently, although neither VRC03 nor F105 was particularly effective. Since the structures of VRC03 and F105 bound to gp120 irrevocably prove their specificity for the CD4bs [14,18], the reasons for weaker sCD4 inhibition are unclear. We also tested the CD4i mab, 17b, as a control. As expected, this mab failed to block sCD4 binding to gp120. [file 1742-4690-9-9-S1.TIFF]
